# Comparative analysis of microarray data in Arabidopsis transcriptome during compatible interactions with plant viruses

**DOI:** 10.1186/1743-422X-9-101

**Published:** 2012-05-29

**Authors:** Olga A Postnikova, Lev G Nemchinov

**Affiliations:** 1 USDA/ARS, Plant Sciences Institute, Molecular Plant Pathology Laboratory, Beltsville, MD, 20705, USA; 2 Institute of Basic Biological Problems, Russian Academy of Sciences, 2 Institute Street, Pushchino, Moscow Region, 142292, Russia

**Keywords:** Arabidopsis, Response to virus infection, Microarray, Co-expressed clusters

## Abstract

**Background:**

At the moment, there are a number of publications describing gene expression profiling in virus-infected plants. Most of the data are limited to specific host-pathogen interactions involving a given virus and a model host plant – usually *Arabidopsis thaliana*. Even though several summarizing attempts have been made, a general picture of gene expression changes in susceptible virus-host interactions is lacking.

**Methods:**

To analyze transcriptome response to virus infection, we have assembled currently available microarray data on changes in gene expression levels in compatible *Arabidopsis-*virus interactions. We used the mean *r* (Pearson’s correlation coefficient) for neighboring pairs to estimate pairwise local similarity in expression in the Arabidopsis genome.

**Results:**

Here we provide a functional classification of genes with altered expression levels. We also demonstrate that responsive genes may be grouped or clustered based on their co-expression pattern and chromosomal location.

**Conclusions:**

In summary, we found that there is a greater variety of upregulated genes in the course of viral pathogenesis as compared to repressed genes. Distribution of the responsive genes in combined viral databases differed from that of the whole *Arabidopsis* genome, thus underlining a role of the specific biological processes in common mechanisms of general resistance against viruses and in physiological/cellular changes caused by infection. Using integrative platforms for the analysis of gene expression data and functional profiling, we identified overrepresented functional groups among activated and repressed genes. Each virus-host interaction is unique in terms of the genes with altered expression levels and the number of shared genes affected by all viruses is very limited. At the same time, common genes can participate in virus-, fungi- and bacteria-host interaction. According to our data, non-homologous genes that are located in close proximity to each other on the chromosomes, and whose expression profiles are modified as a result of the viral infection, occupy 12% of the genome. Among them 5% form co-expressed and co-regulated clusters.

## Background

Viruses are among the most agriculturally important groups of plant pathogens, causing serious economic losses in many major crops by reducing yield and quality [1]. Although viruses have relatively simple genetic structure, the detailed mechanisms of their interaction with host plants and means by which they manipulate a plant’s physiology toward their needs and trigger antiviral responses in hosts are still not well-defined [2-5]. Among the most important consequences of viral pathogenesis are changes in expression of host genes that define both the fate of the virus and the host’s survival chances. If plants are capable of efficiently fighting infections by inherited genetic tools, such as resistance (*R*) genes that are abundant in every plant species [6], they immediately initiate general resistance pathways leading to a hypersensitive response (HR). In susceptible plants lacking *R* genes to a specific viral pathogen, viruses induce a variety of responses to prime and elevate their infections. These include expression changes associated with cellular processes redirected by viruses for their demands and host defensive reactions to the pathogenesis [3]. Understanding the balance and interplay between these two types of responses would bring light to poorly characterized molecular mechanisms of viral comprehensive control of host immune system and to the counteracting host signaling pathways. It will also help to explain continuous and interconnected genetic variability in viral and host populations, that is, co-evolution of plants and viruses.

At the moment, there are a number of publications describing gene expression profiling in virus-infected plants that are derived mostly from DNA microarrays. They indicate a significant impact of viral infection on a wide array of cellular processes [7]. Usually, altered functional categories include responses to biotic and abiotic stresses, changes in basal plant metabolism, protein synthesis, developmental and photosynthetic processes [7-10].

Most of the data are limited to specific host-pathogen interactions involving a given virus and a model host plant, which usually is *Arabidopsis thaliana.* In spite of several efforts to summarize general changes in plant gene expression (due to viral, bacterial and fungal infections, insect attack, other biotic and abiotic stresses) having been made [3-5,11], a general picture of gene expression changes in susceptible virus-host interactions is missing. Detailed knowledge about the groups of host genes participating in and/or responsive to viral pathogenesis may lead to new assertions on how host cells are controlled by infection, which defense and stress mechanisms are deployed, and why disease symptoms or deviation from normal in the growth of a plant are developed [1,5].

In this work, in order to analyze transcriptome response to virus infection, we have assembled currently available microarray data on changes in gene expression levels in compatible *Arabidopsis*-virus interactions and attempted to create a functional classification of the genes with altered expression levels. We conclude that each virus-host interaction is unique in terms of the genes with altered expression levels and the number of shared genes affected by all viruses is very limited. Importantly, we also demonstrate that responsive genes may be grouped or clustered based on their co-expression pattern and chromosomal location.

## Methods

### Data source, microarray data

*Arabidopsis* expression data were obtained from Nottingham Arabidopsis Stock Centre microarray (NASC), ArrayExpress from the European Bioinformatics Institute database and the Gene Expression Omnibus database. Additional data were retrieved from supplementary material of published papers [see Additional file 1]. The data sets were log transformed (when needed) and significant genes were selected according to *P <* 0.05. We selected only those genes that significantly changed their expression level in response to pathogen attack by at least two-fold. Tandem duplicates were removed from the resulting profile. The total number of collected genes across all experiments was 52488. These data represent 44 experiments with 3 different types of pathogens: virus, bacteria and fungus. Among them there were 11 viruses and the total number of genes with significantly altered expression elicited by these viruses was 16816. This number included many identical genes (with the same ID) recorded in different experiments. After subtraction of the repeating genes, a list of 7639 unique genes was obtained. The same data set was used to obtain data for bacteria 17734 (11409 unique genes) and 15426 for fungi (among them 11047 unique genes).

### Data analysis

We performed a meta-analysis of all the collected data on compatible virus-host interactions and also on the whole database representing viral, bacterial and fungal interactions with the host plant. We used tools from TAIR to search GO annotations and functionally classify *Arabidopsis* genes. To find over-represented functional groups among activated or repressed genes during virus-host interactions we used Babelomics 4 FatiGO [12] and SAE from agriGO [13]. To visualize this data we used REViGO software [14].

### Clustering pathogen related genes

The level of co-expression between two genes was defined as the Pearson’s correlation coefficient (*r*) of the expression level for these genes. To test for pairwise local similarity in expression in the *Arabidopsis* genome, the mean *r* of the expression profiles for neighboring pairs of genes was calculated [15].

The mean *r* calculated from the real data set was then compared with the mean *r* calculated from 1000 data sets in which the order of genes in the *Arabidopsis* genome was randomized. We generated the stochastic distribution using a function that generates an even distribution of stochastic numbers. The proportion of genes found in clusters and the size distribution of clusters were calculated, and the values were averaged for 1000 iterations.

## Results and discussion

### Broad changes in gene expression during susceptible virus-host interactions

To analyze plant response to virus infection, we have assembled currently available microarray data on changes of gene expression levels in *Arabidopsis thaliana* in response to infection with various plant viruses: *Cabbage leaf curl virus* (CalCuV), *Cauliflower mosaic virus* (CAMV)*, Cucumber mosaic virus* (CMV)*, Lettuce mosaic virus* (LMV)*, Plum pox virus* (PPV), *Turnip crinkle virus* (TCV*), Tobacco etch virus* (TEV), *Tobacco mosaic virus* (TMV and TMV-Cg), *Tobacco rattle virus* (TRV), *Turnip mosaic virus* (TuMV) and *Oilseed rape mosaic tobamovirus* (ORMV) [7,8,10,16-22].

**Figure 1:**
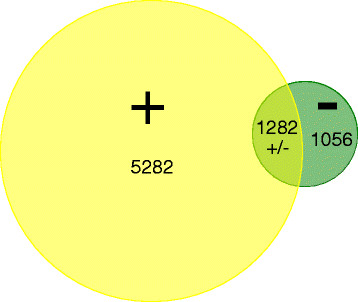
**Venn diagram depicting the distribution of 7639 unique genes in response to infection with different viruses.** The yellow circle represents induced genes and the green circle represents repressed genes. The radius of each circle corresponds to the number of genes in the group of induced or repressed genes.

The total number of genes in the assembled experiments with significantly altered expression elicited by these viruses was 16816. Among them 8684 were upregulated and 8132 were downregulated (a threshold of at least 2-fold change in expression level). However, this number included many identical genes (with the same ID) recorded in different experiments. After subtraction of the repeated genes, a list of 7639 unique genes was obtained [see Additional file 2], which represents 23% of the whole *Arabidopsis* genome. These are the genes either needed for the host to defend itself against the virus or for the virus to re-arrange host cellular machinery for its own needs. More than two thirds of these genes (69%) were always upregulated and only 13% were always downregulated. A sizeable portion of the genes (17%) had differential expression in response to infection with different viruses (Figure 1). Thus, the total number of induced genes (5282) exceeds that of repressed genes (1056) more than five-fold in our reduced (unique IDs) database. Approximately 15.5% of responsive genes had previously been described as involved in plant defense.

Does the larger number of activated genes as compared to repressed genes correspond to a general trend of plant response to virus, reflecting a greater diversity of upregulated genes? In other words, based on this information, can we conclude as other authors have done [23], that there is a widespread induction of the host’s biological processes due to the virus infection? *De facto*, it depends on several conditions. First, individual databases available for different viruses differ extensively in the number of repressed or induced genes and combined analysis is greatly influenced by this ratio in the most comprehensive databases. Second, as mentioned above, the pool of upregulated genes is larger because of the greater variety of affected genes whereas the quantity of downregulated genes is limited. Otherwise stated, more diverse genes are upregulated during different virus infections whereas downregulated genes tend to be common regardless of the particular virus. Lastly, a more accurate illustration of the general status of gene expression changes can be derived from the analysis of their proportional representation in the sets of induced or repressed genes within each functional category, which is the subject of the following section.

Using TAIR’s functional categorization, we first assigned each gene to one of the three main gene ontologies (GO) - Biological Process, Cellular Components and Molecular Function, and next to a specific functional category (FC). It is important to emphasize three key points when relying on the GO terms in analyzing expression profiles: their generality, their obvious redundancy and their incompleteness. Redundant annotations and multiple descriptions of the same biological mechanisms represent special concern undermining an effort to address consistency in characterization of gene products. Still, the Gene Ontology project [24] currently provides the most constructive way to find functionally equivalent terms for the purpose of classifying gene product properties.

**Figure 2:**
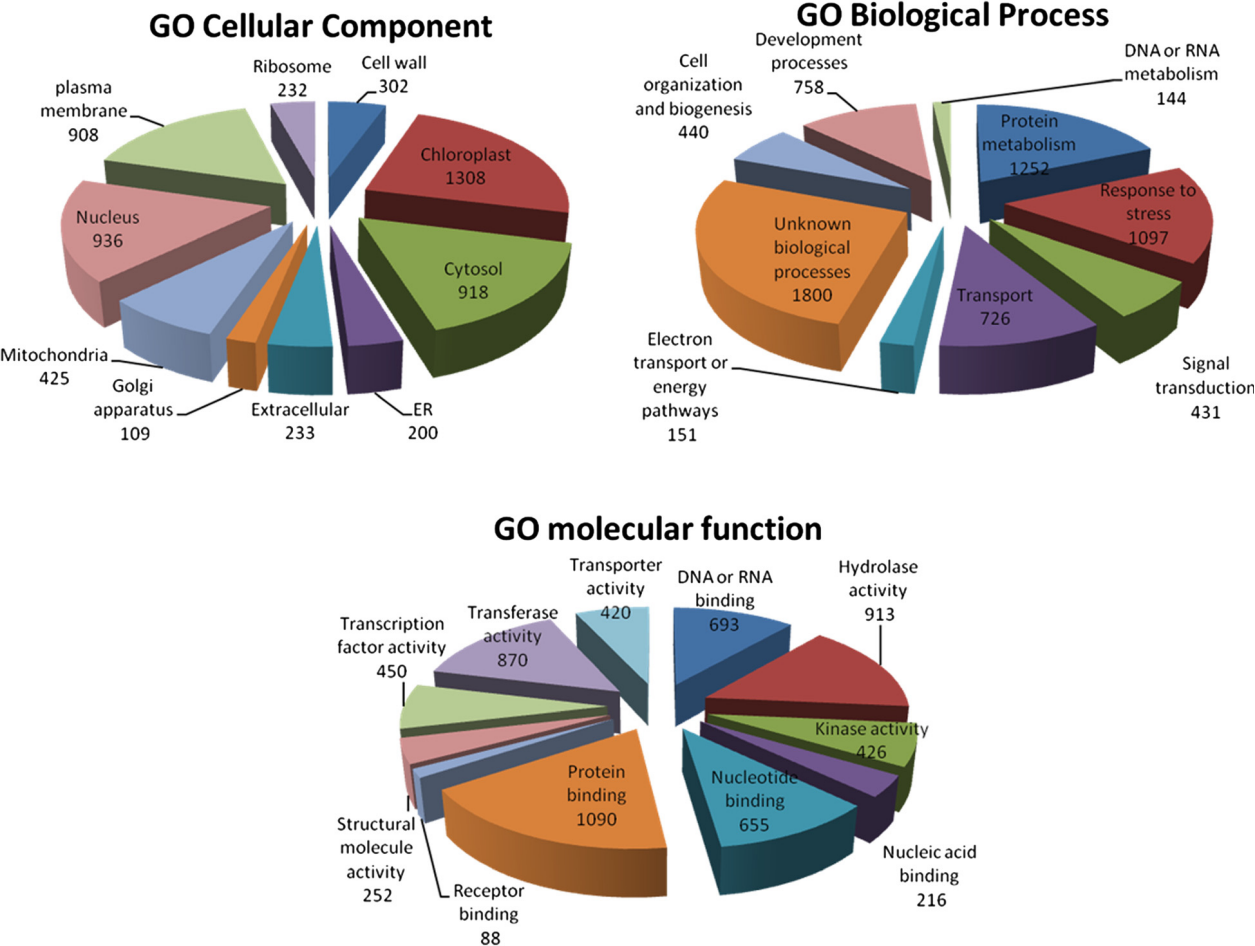
Distributions of the responsive genes by FC in each given GO domain of the assembled viral database.

Figure 2 and Table 1 show distributions of the responsive genes in different FC with respect to the total number of genes in each given GO domain of the assembled viral database. Noticeably, some of the key functions with a large number of affected genes (upregulated- or downregulated) are in the following categories: chloroplast (21% of total genes in FC), nucleus (15%) and cytosol (15%) in GO Cellular Components; hydrolase and transferase activity (13% both), protein and DNA or RNA binding (16% and 10%, respectively) in GO Molecular Function; protein metabolism (18%) and response to stress (16%) in GO Biological Process.

**Table 1:** Distributions of genes in the three main GO domains for the whole genome and assembled viral database

	**Number of genes in genome**	**% of GO domain**	**Number of genes in virus database**	**% of virus database**	**Ratio % of virus/% of GO domain**
**GO Cellular Component**					
**cell wall**	**605**	**2.42**	**302**	**4.94**	**2.04**
**chloroplast**	**2941**	**11.78**	**1308**	**21.39**	**1.81**
**cytosol**	**1662**	**6.66**	**918**	**15.01**	**2.25**
**ER**	**425**	**1.70**	**200**	**3.27**	**1.92**
**extracellular**	**454**	**1.82**	**233**	**3.81**	**2.09**
**Golgi apparatus**	**249**	**1.00**	**109**	**1.78**	**1.79**
**mitochondria**	**1123**	**4.50**	**425**	**6.95**	**1.54**
**nucleus**	**2504**	**10.03**	**936**	**15.30**	**1.53**
**other cellular components**	**4405**	**17.65**	**1056**	**17.27**	**0.98**
**other cytoplasmic components**	**3384**	**13.56**	**1695**	**27.71**	**2.04**
**other intracellular components**	**4310**	**17.27**	**1914**	**31.29**	**1.81**
**other membranes**	**3373**	**13.52**	**1358**	**22.20**	**1.64**
**plasma membrane**	**1862**	**7.46**	**908**	**14.85**	**1.99**
**ribosome**	**472**	**1.89**	**232**	**3.79**	**2.01**
**unknown cellular components**	**9632**	**38.60**	**1024**	**16.74**	**0.43**
**Total**	**24956**	**100.00**	**6116**	**100.00**	
**GO Molecular Function**					
**DNA or RNA binding**	**2894**	**10.59**	**693**	**10.03**	**0.95**
**hydrolase activity**	**2959**	**10.82**	**913**	**13.22**	**1.22**
**kinase activity**	**1342**	**4.91**	**426**	**6.17**	**1.26**
**nucleic acid binding**	**1467**	**5.37**	**216**	**3.13**	**0.58**
**nucleotide binding**	**2114**	**7.73**	**655**	**9.48**	**1.23**
**other binding**	**4529**	**16.57**	**1258**	**18.21**	**1.10**
**other enzyme activity**	**3200**	**11.70**	**1157**	**16.75**	**1.43**
**other molecular functions**	**1003**	**3.67**	**311**	**4.50**	**1.23**
**protein binding**	**2426**	**8.87**	**1090**	**15.78**	**1.78**
**receptor binding or activity**	**271**	**0.99**	**88**	**1.27**	**1.29**
**structural molecule activity**	**536**	**1.96**	**252**	**3.65**	**1.86**
**transcription factor activity**	**1681**	**6.15**	**450**	**6.52**	**1.06**
**transferase activity**	**2509**	**9.18**	**870**	**12.60**	**1.37**
**transporter activity**	**1266**	**4.63**	**420**	**6.08**	**1.31**
**unknown molecular functions**	**10851**	**39.69**	**1651**	**23.90**	**0.60**
**Total**	**27340**	**100.00**	**6907**	**100.00**	
**GO Biological Process**					
**cell organization and biogenesis**	**1245**	**4.44**	**440**	**6.23**	**1.40**
**developmental processes**	**2309**	**8.24**	**758**	**10.73**	**1.30**
**DNA or RNA metabolism**	**444**	**1.58**	**114**	**1.61**	**1.02**
**electron transport or energy pathways**	**294**	**1.05**	**151**	**2.14**	**2.04**
**other biological processes**	**2157**	**7.70**	**965**	**13.66**	**1.78**
**other cellular processes**	**12254**	**43.72**	**3950**	**55.93**	**1.28**
**other metabolic processes**	**12875**	**45.93**	**4092**	**57.94**	**1.26**
**protein metabolism**	**4256**	**15.18**	**1252**	**17.73**	**1.17**
**response to abiotic or biotic stimulus**	**2175**	**7.76**	**1037**	**14.68**	**1.89**
**response to stress**	**2424**	**8.65**	**1097**	**15.53**	**1.80**
**signal transduction**	**1366**	**4.87**	**431**	**6.10**	**1.25**
**transport**	**2080**	**7.42**	**726**	**10.28**	**1.39**
**unknown biological processes**	**11282**	**40.25**	**1800**	**25.48**	**0.63**
**Total**	**28031**	**100.00**	**7063**	**100.00**	

It was essential to determine the extent of involvement of specific FC that represents groups of genes implicated in a particular biological mechanism in the host reaction to infection. Therefore we compared the distribution of genes assigned to different FC on the whole genome of *Arabidopsis* with the corresponding distribution within our database of genes that are involved in response to virus infection (Table 1), [see Additional file 3]. Presumably, the greater the share each category occupies in the virus database versus in the whole genome, the greater this FC participates in host response. We found that the percentage of genes covered by several categories, such as cell wall, cytosol, extracellular, ribosome, electron transport or energy pathways, was twice as much as the normal distribution in the whole genome, thus emphasizing the important role of these functions in host-viral interactions. A share of genes in the FC “response to stress” was also 1.7 times higher in the viral database as compared to the whole genome (Table 1) and[see Additional file 3].

### Common responses to different viruses

To find common responses to different viruses, we compared patterns of gene response in individual susceptible interactions. In order to do this, we used the number of shared genes among every pair of viruses to compute a similarity matrix between them according to the formula *S*_*ij*_ = 2*n*_*ij*_/(*n*_*i*_ + *n*_*j*_), where *n*_*i*_ and *n*_*j*_ are the number of genes with altered expression level belonging to the database for virus *i* and virus *j*, respectively, and *n*_*ij*_ represents the number of genes shared between both viruses [9]. Next, we arranged the computed data in accordance with the value of *S*_*ij*;_ the higher this value is, the more similarity that exists between the two compared virus-host interactions.

**Figure 3:**
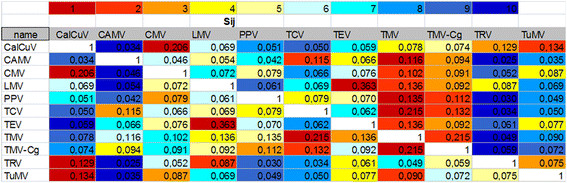
**Similarity matrix (*S***_***ij***_**) reflecting changes in the *Arabidopsis*transcriptome in response to different *Arabidopsis*-virus interactions.** The higher the value of the *S*_*ij*_, the more similarity exists between two compared virus-host interactions. Each cell represents an individual virus-host interaction *S*_*ij*_, color-delineated according to the level of similarity ranging from considerable (red) to weak (dark blue).

As presented in Figure 3, changes of the expression pattern in response to infection with the majority of viruses were similar to the ones associated with TMV and TMV-Cg. Responses to TEV and LMV potyviruses also showed significant similarity to each other. On the other hand, even responses to RNA versus DNA viruses can be quite similar as exemplified by CMV and CalCuV.

Although the number of common genes affected by all viruses is very limited, each virus-host interaction is unique in terms of which genes have altered expression levels. Among them are several pathogenesis-related (PR) genes, albeit they seem to be specifically upregulated in response to particular viruses: CalCuV, TMV and TEV (PR1), CMV, CalCuV, TMV and TRV (PR2 and PR4), LMV and TRV (PR5), CMV and TMV (PR3).

Overall, we have found only 198 genes that frequently change their expression in response to the majority of the viruses [see Additional file 4. Those include genes participating in the defense or immune pathways as well as genes of catabolic and regulation processes. One of the most frequently induced genes in all interactions is AT5G38530 of the tryptophan biosynthetic pathway. The tryptophan pathway provides precursors for the synthesis of key secondary metabolites such as auxin, indole-3-acetic acid (IAA), and other molecules that help protect plants against pathogens and herbivores [25].

### Proportional representation of different functional categories in the sets of induced or repressed genes

To identify overrepresented functional groups among activated or repressed genes, we subjected genes to analysis by Babelomics 4 FatiGO [12] and SEA from agriGO [13]. FatiGO uses Fisher’s exact test for 2 × 2 contingency tables to scan for significant over-representation of GO terms in one set with respect to the other. Singular enrichment analysis (SEA) identifies enriched GO terms in a list of microarray probe sets or gene identifiers. Using both FatiGO and SEA ensures finding accurate, condensed biological data by comparing a query list to a background population from which it is derived [12]. That is, such analyses predict a role of a certain biological processes in total response to infection rather than merely calculate a number of upregulated and downregulated genes.

When implemented with the assembled data set, FatiGO and SEA identified over-represented FC and subcategories among the sets of induced or repressed genes belonging to each of the main GO domains. Overrepresented in the set of repressed genes were those involved in defense response, hormone signaling (JA, ABA), response to external stimulus, photosynthesis and bioenergetics processes (encoding photosystem I and II proteins and electron transport chain) [see Additional file 5. Downregulation of these functions is presumably due to the virus overtaking host defense-related pathways and causing physiological changes associated with the disease symptoms [22].

Overrepresented in the set of induced genes were those participating in response to abiotic stimulus, responses to organic and inorganic substances, nitrogen component metabolic processes and protein transport (Golgi vesicle transport, protein targeting, and cytoskeletal protein binding). The latter host pathways are essential for facilitating virus intracellular movement. Another example of a biological process that was found only in the upregulated gene set is chromatin organization (histone modification). Chromatin structural features and posttranslational modifications play a crucial role in the regulation of gene expression [26]. Epigenetic ‘marks’ generated by modifications of histones and DNA are spread over vast regions of chromosomes and can be altered in response to stress.

Assembling information from multiple sources, such as different microarray platforms, experimental conditions, stages of infection when samples were collected, etc. raises a question of the integrity of the combined data, since it is hardly possible to eliminate the “batch effect” from influencing final results. Even so, these disparities are not likely to change the biological truth. For instance, as presented in Figure 3, the values for TMV and TMV-Cg (a crucifer-infecting strain of TMV) are very similar to each other even though they were obtained by different authors using different platforms in totally different environments. To take into account some of the determining factors (such as infection stages at the time of analyses), when different genes may become activated and/or repressed, we also looked into combined microarray data on early and late responses to virus infection and analyzed it as much as statistically possible.

**Figure 4:**
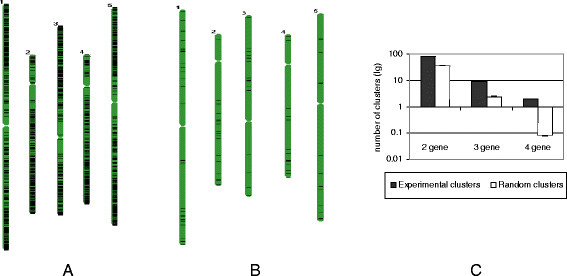
**Overrepresented GO terms in induced and repressed gene sets in the early and late stages of infection.** The ontology table displays significant GO categories in response to infection with a compatible virus as determined by Babelomics 4 FatiGO and agriGO SEA. Box color reflects the flash discovery rate (FDR) to which the given GO category belongs, as shown on the scale.

Applying agriGO tools, we found that early, non-symptomatic, phases of infection are characterized by massive induction of genes belonging to both common and stress-responsive pathways. Overrepresented in the set of activated genes were amine biosynthetic processes, aromatic amino acid family metabolic processes, photosynthetic activity and responses to biotic and abiotic stresses (Figure 4). Late stages of pathogenesis, when plants are systemically infected, are characterized by repression of the majority of the stress-responsive pathways activated at the early phases, such as response to abscisic acid stimulus, response to wounding, innate immune response, response to oxidative stress, response to auxin stimulus, callose deposition in cell wall and glycosinolate metabolic process.

One of the main features of the late host response is repression of photosynthetic and energy pathways: photosystem I and II assembly, pentose-phosphate cycle, etc. (Figure 4). Chlorosis, or yellowing of normally green plant tissue, because of the disruption of chloroplast structure and function and a decreased amount of chlorophyll is often a direct result of these deficiencies. On the contrary, cellular respiration, catabolic processes, proteolysis and senescence are overrepresented in the induced gene set at the late stages of virus infection. Among the common characteristics of both the early and late responses is negative regulation of developmental processes. In essence, sets of host genes affected at the late stage of infection closely resemble the general picture of gene expression changes caused by viral pathogenesis.

### Pathogenesis-related two- to four-gene clusters in the genome of *Arabidopsis thaliana*

**Figure 5:**
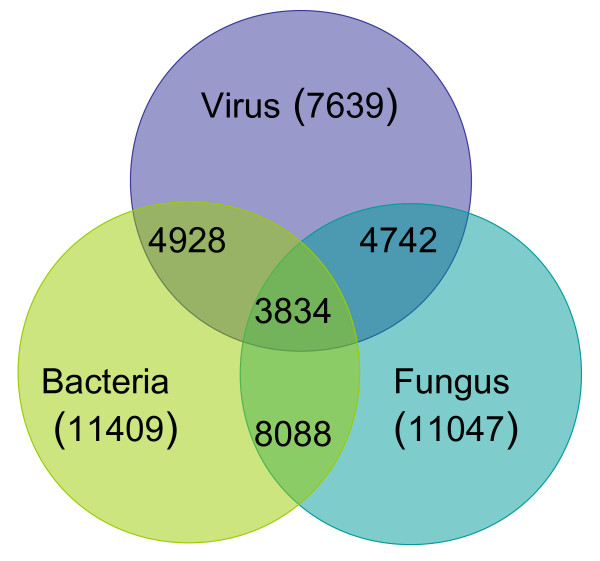
**Clusters of non-homologous, defense-related genes in the genome of*A*.*thaliana.*(A)** Chromosomal distribution of 1594 groups of neighboring genes, which responded differentially (repressed or activated) to various viral infections. **(B)** Distribution of pathogen-related, co-expressed clusters on the chromosomes of *A*. *thaliana*. **(C)** Comparison between the number of clustered genes obtained from the random distribution data set or found experimentally. The height of the bars represents the number of defense-related gene clusters of the corresponding size either revealed in the experiment (black columns) or estimated by stochastic distribution (unfilled columns).

While looking at the data derived from the analysis of publicly available microarray repositories, we noticed that genes with expression profiles modified as a result of viral infection were often (12% of genome) located in close proximity to each other on the same chromosomes. Analyzing only close proximity and differential response to the infection (repressed or activated genes) we discovered 1594 such groups of genes (Figure 5A). Among them were 5 groups consisting of 8 genes, 7 groups of 7 genes, 20 groups of 6 genes, and 35 groups of 5 genes. Assuming that the order of genes with altered expression patterns along the chromosomes is not accidental [27] but reflects their functional role, we hypothesized that these groups of neighboring genes distributed across the *Arabidopsis* genome may further be divided into co-regulated and co-expressed blocks of genes or clusters. Since microarray data sets on susceptible host-virus interactions were not large enough to statistically predict clusters of genes with similar expression changes, we combined them with data from microarray experiments representing bacterial-host and fungal-host interactions and then used “viral sets” as a base for filtering out only analogous genes. This way, we were able to compose groups of co-expressed genes.

We found 207 neighboring co-expressed genes which fall into 98 clusters under conditions of pathogenesis [see Additional file 6]. These clusters consist of groups of physically linked and functionally related genes (response to pathogen) that are co-expressed (correlation coefficient *r* ≥ 0.7) and possibly co-regulated but share no sequence homology. Although most of them were differentially expressed, 22 clusters were always upregulated and only 2 clusters were always downregulated. Among all identified clusters only two contained four genes, nine were composed of three genes, and eighty-six contained two genes (Figure 5B).

Differences between the stochastic distribution and the actual distribution revealed in this experiment were observed both in the number of genes included in clusters and in cluster size (Figure 5C). The number of two-gene and three-gene clusters in the experiment was almost 2.5 times and 4 times higher, respectively, than expected by chance. Four-gene clusters were obtained in the experiment only; clusters of this size were not predicted to form by chance. We found 16 overlapping clusters between our pathogen-response clusters and those predicted by Zhan et al. using microarray data representing 128 experimental conditions [28]. Apparently, genes forming these clusters are broadly co-expressed in a wide range of conditions.

To find out if there are any functional relationships between locally co-expressed genes, we used TAIR’s GO for *Arabidopsis*. We found 7 molecular functions, which are shared for each of 8 gene pairs as well as 13 cellular components that are common for 23 gene pairs. As revealed by the AraCyc database files from the Plant Metabolic Network, none of our clusters belongs to the same pathway [see Additional file 6].

Therefore, co-expressed neighbors do not seem to be associated with a particular GO [29]. However, it does not mean that clustered genes are not *functionally* related to each other. That is, in spite of belonging to different GO categories, co-expressed groups of genes are affiliated with the same function – stress response. Plants re-arrange their metabolism upon recognition of pathogen-associated molecular patterns (PAMP) so that genes of different GO categories that are involved in defense mechanisms are engaged [30].

Interestingly, one of the clusters includes three genes encoding leucine-rich repeat (LRR) family proteins: AT1G33590, AT1G33600 and AT1G33610. Two more genes that are absent in available microarray data sets, AT1G33612 and AT1G33670, also encode LRR proteins and are located in the same chromosomal region. In addition, we found three other clusters containing genes with common domain structure and functional characteristics: i. cluster with a Toll-Interleukin-Resistance (TIR) domain (AT1G72900 and AT1G72910); ii. cluster with genes encoding SAM superfamily proteins (S-adenosyl-L-methionine-dependent methyltransferases superfamily, AT4G00740, and AT4G00750); and iii. cluster with genes encoding histone superfamily proteins (AT4G40030 and AT4G40040).

Previously, we reported on the clustering of pathogen-response genes in the genome of *Arabidopsis thaliana*[31]. That study was based on the profiling of EST databases derived from different plant species infected with fungi, bacteria, and viruses [32]. While comparing gene clusters revealed by broad EST mining with analysis of microarray data sets specific for compatible virus-host interactions (this investigation), we found that most groups of neighboring genes determined in the former study could be included in the clusters identified in this work, providing that both up and downregulated genes derived from different experiments are counted (Figure 5A). However, if only co-expressed genes are considered (Figure 5B), overlap between these two data sets is quite low, which could possibly be explained by a unique pattern of chromosomal gene clustering characteristic for different types and/or individual pathogens.

### Common genes participate in virus-, fungi- and bacteria-host interaction

As mentioned above, we tried to assemble the largest possible database of *Arabidopsis* genes responsive to viral infections using currently available microarray data. For comparison, we also put together genes derived from microarray experiments with bacteria and fungi. Since there is significantly more information on changes in plant gene expression due to infection with bacteria and fungi, we limited the number of genes to approximately the same number as was compiled for viruses: 17734 for bacteria (11409 unique genes) and 15426 for fungi (among them 11047 unique genes) [11,33-36].

**Figure 6:**
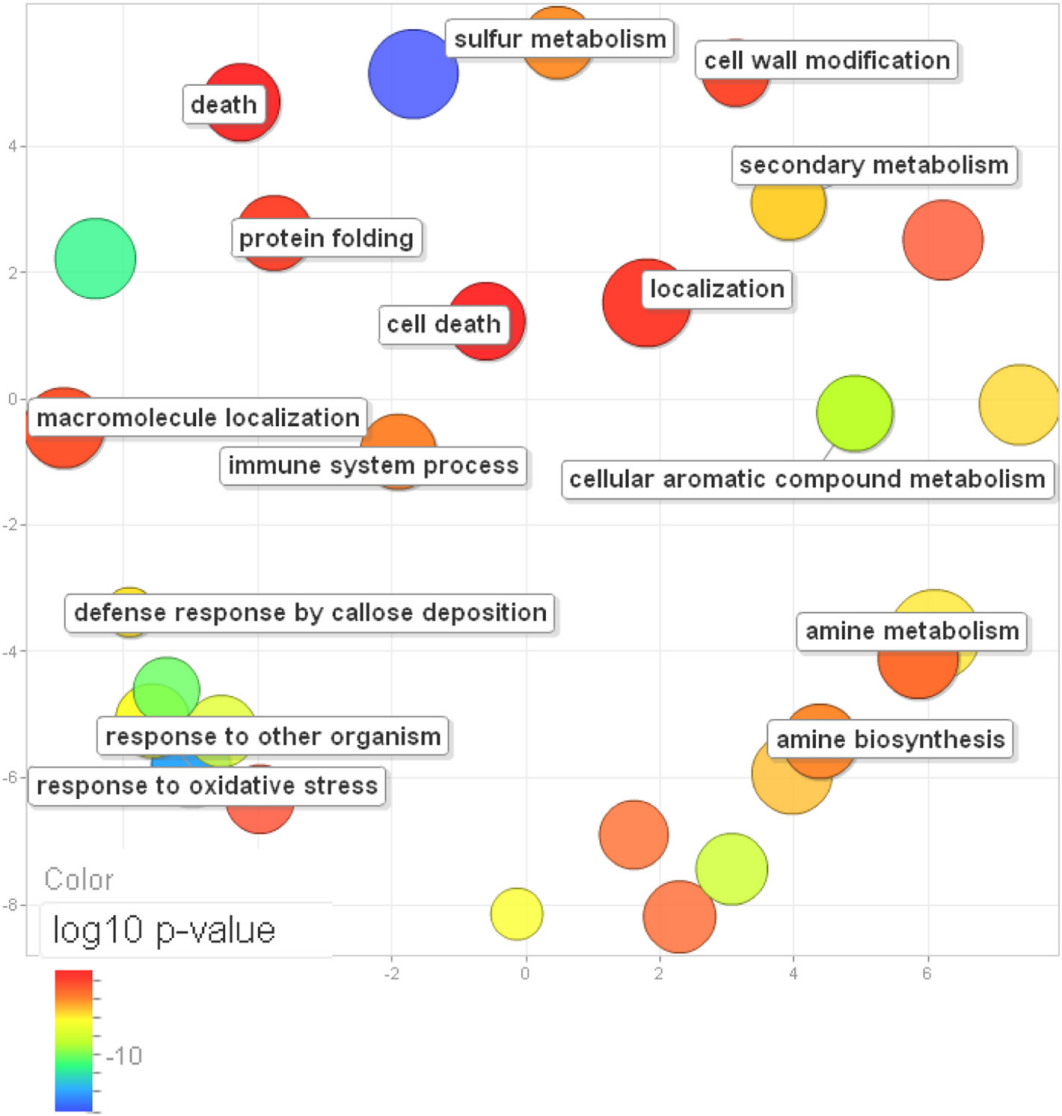
**Venn diagram depicting similarity between responses to viral, bacterial and fungal pathogens.** The numbers in parentheses represent a number of unique genes responsive to a particular type of pathogen.

 Gene expression changes in response to all pathogens were very similar. In spite of specific interactions between host plants and each of the pathogens, nearly half of the genes associated with viral infections in susceptible hosts were also involved in response to bacterial or fungal infections (Figure 6). Next, we selected genes which were induced or repressed in all three types of interactions [see Additional file 7]. Most of these genes belong to co-expressed chromosomal regions, or clusters: there were 79 two-gene clusters and 4 three-gene clusters. This suggests that common genes participating in response to biotic stress may be co-regulated and organized in clusters. A small cluster containing non-homologous genes AT1G20100 and AT1G20110 is especially interesting since it is engaged in response to the majority of plant viruses. One of these genes encoding a RING/FYVE/PHD zinc finger superfamily protein participates in signal transduction pathways and another one is a protein of unknown function.

**Figure 7:**
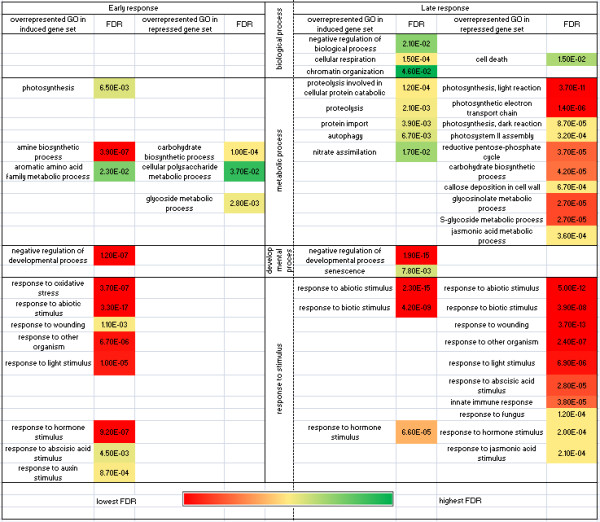
**Scatter plot of over-represented GO terms from upregulated gene sets of viral, bacterial and fungal pathogens.** GO terms were obtained using the SAE tool from agriGO and data were visualized by REViGO. The axes have no intrinsic meaning; the guiding principle is that semantically similar GO terms remain close together in the plot. Color scale indicates log10 *p*-value (red is higher and blue is lower). Disc size is proportional to the log number of genes in the category.

While determining significantly over-represented functional groups that include genes activated during virus-host interactions, we have also found groups of genes whose biological functions are common for all three pathogens (virus, bacteria, and fungus) regardless of whether there is a susceptible or resistant type of interaction. To obtain significantly over-represented GO terms from gene sets upregulated during infection with these pathogens, we used the SAE tool from agriGO [13]. In order to visualize data we used REViGO [14]. We found that some of the GO categories, such as sulfur metabolism, cellular aromatic compound metabolism, and cell wall modification were enriched in the downregulated genes under compatible virus infections. However, when we considered resistant-type interactions of bacteria and fungi with host plants, we found that the same GO categories were enriched with activated genes (Figure 7). In addition, when genes involved in general immune responses were analyzed using SAE and REViGO in both susceptible and resistant types of interactions, they were also found to be induced. Unfortunately, the limited amount of microarray data did not allow a full-scale comparison between susceptible and resistant types of interactions, which would be useful in terms of understanding the mechanisms of *R* gene-mediated resistance.

## Conclusions

We have assembled currently available microarray data on changes in gene expression levels in compatible *Arabidopsis-*virus interactions. In summary, we found that there is a greater variety of upregulated genes in the course of viral pathogenesis as compared to repressed genes. Distribution of the responsive genes in combined viral databases differed from that of the whole *Arabidopsis* genome, thus underlining a role of the specific FC in common mechanisms of general resistance against viruses and in physiological/cellular changes caused by infection. Using integrative platforms for the analysis of gene expression data and functional profiling, we identified overrepresented functional groups among activated and repressed genes, which provided an in-depth view of the role of certain biological processes in response to infection. Each virus-host interaction was found to be unique in terms of the genes with altered expression levels, and the number of common genes affected by all viruses was very limited. We discovered that genes with expression profiles modified as a result of viral infection were often located in close proximity to each other on the same chromosomes forming a multiple clusters, consisting of physically linked and functionally related genes. Finally, combining genes derived from microarray experiments with bacteria and fungi with a viral data set, we observed that gene expression changes in response to all pathogens were very similar and that nearly half of the genes associated with viral infections in susceptible hosts were also involved in response to bacterial or fungal infections.

## Abbreviations

HR, Hypersensitive response; CalCuV, Cabbage leaf curl virus; CAMV, Cauliflower mosaic virus; CMV, Cucumber mosaic virus; LMV, Lettuce mosaic virus; PPV, Plum pox virus; TCV, Turnip crinkle virus; TEV, Tobacco etch virus; TMV and TMV-Cg, Tobacco mosaic virus; TRV, Tobacco rattle virus; TuMV, Turnip mosaic virus; ORMV, Oilseed rape mosaic tobamovirus; GO, Gene ontology annotations; ET, Ethylene; JA, Jasmonic acid; ABA, Abscisic acid.

## Competing interests

The authors declare that they have no competing interests.

## Authors’ contributions

OP collected and analyzed the data. LN wrote the manuscript. All authors discussed the results and commented on the manuscript. All authors read and approved the final manuscript.

## Supplementary Material

Additional file 1 Microarray Sources. Click here for file

Additional file 2 A list of unique genes participating in response to virus infection. Click here for file

Additional file 3 Comparison of % coverage of different FC between whole genome and virus responsive genes in the assembled database. Click here for file

Additional file 4 Over-represented GO in response to susceptible virus interactions. Click here for file

Additional file 5 Common genes in response to susceptible virus interactions. Click here for file

Additional file 6 Cluster genes. Click here for file

Additional file 7 Common genes in response to three pathogens. Click here for file
